# Fluoxetine Reduces Murine Graft-Versus-Host Disease by Induction of T cell Immunosuppression

**DOI:** 10.1007/s11481-013-9463-7

**Published:** 2013-05-04

**Authors:** Veerle Gobin, Katleen Van Steendam, Sabine Fevery, Kelly Tilleman, An D. Billiau, Damiaan Denys, Dieter L. Deforce

**Affiliations:** 1 Laboratory of Pharmaceutical Biotechnology, Faculty of Pharmaceutical Sciences, University of Ghent, Harelbekestraat 72, 9000 Ghent, Belgium; 2 Laboratory of Experimental Transplantation, University of Leuven, Leuven, Belgium; 3 Department of Psychiatry, Academic Medical Centre, University of Amsterdam, PA.2-179, Meibergdreef 5, 1105 AZ Amsterdam, The Netherlands; 4 The Netherlands Institute for Neuroscience, an Institute of the Royal Netherlands Academy of Arts and Sciences, Meibergdreef 47, 1105 BA Amsterdam, The Netherlands

**Keywords:** Serotonin reuptake inhibitors, Fluoxetine, Graft-versus-host disease, Immunosuppression, T cells

## Abstract

Serotonin reuptake inhibitors (SRIs) are widely used drugs in the treatment of depression and anxiety disorders. Although SRIs are generally regarded as safe drugs with relatively few side effects, literature suggests that high concentrations of SRIs may alter immune function. We investigated whether high-dose treatment with fluoxetine was able to suppress acute graft-versus-host disease (GvHD) in a MHC-matched, minor histocompatibility antigen mismatched murine bone marrow transplantation model. We found that high doses fluoxetine induce a significant reduction of clinical symptoms and increase survival of these animals. The amelioration of clinical GvHD was accompanied by a reduced expansion of alloreactive T cells. We further analyzed the direct in vitro effect of six SRIs on the viability and proliferation of human T cells and found an anti-proliferative and pro-apoptotic effect that was significantly larger in activated than in resting T cells. We discuss these results in the light of potential future exploration of SRIs as a novel class of T cell immunosuppressive drugs.

## Introduction

Serotonin reuptake inhibitors (SRIs) belong to the most frequently prescribed drugs worldwide. While originally introduced to treat major depressive disorder, they have proven to be effective in a number of psychiatric and neurological conditions such as obsessive-compulsive disorder, panic disorder and generalized anxiety disorder (Hughes et al. 1999; Vaswani et al. 2003). In the past decades, it has become clear that SRIs not only affect biological mechanisms within the central nervous system, but also have an influence on immunity. Evidence exists that SRIs may attenuate autoimmune responses in experimental autoimmune encephalomyelitis, collagen-induced arthritis, murine allergic asthma and contact hypersensitivity reaction (Vollmar et al. 2009; Taler et al. 2011; Yuan et al. 2012; Sacre et al. 2010; Roumestan et al. 2007; Kubera et al. 2012). Hypothesizing that SRIs may hold potential as a novel class of immunosuppressive drugs, the first aim of this study was to determine whether SRIs could also suppress alloreactive T cell responses in murine graft-versus-host disease (GvHD). Using a MHC-matched, minor histocompatibility antigen (miHA)-mismatched model of allogeneic bone marrow transplantation (BMT), we deliver proof-of-concept evidence that SRIs may also attenuate murine GvHD.

The immunological alterations induced by SRIs in animal models of disease are reflected in the direct effects these drugs exert on lymphocytes. Several in vivo and in vitro reports have demonstrated a negative effect of SRIs on mitogen-induced lymphocyte proliferation (Berkeley et al. 1994; Edgar et al. 1998; Edgar et al. 1999; Pellegrino and Bayer 1998, 2000; Taler et al. 2008; Fazzino et al. 2008), pro-inflammatory cytokine secretion (Taler et al. 2008; Taler et al. 2007; Xia et al. 1996; Maes et al. 1999; Kubera et al. 2001) and lymphocyte viability (Taler et al. 2008; Taler et al. 2007; Xia et al. 1997). Although it is clear that several research groups have investigated the anti-proliferative and pro-apoptotic effects of SRIs, variation in the SRIs studied, the concentrations used and the experimental read-out hampers comparison between studies and interpretation of results. Therefore, a comprehensive study comparing the anti-proliferative and pro-apoptotic effects of all available SRIs in both activated and resting human lymphocytes would contribute to our understanding of the potential immunomodulatory effects of SRI treatment. Thus, the second aim of this study was to determine and compare the direct in vitro effects of six different SRIs used in clinical practice (paroxetine, fluoxetine, sertraline, fluvoxamine, citalopram and venlafaxine) on the viability and proliferation of lymphocytes from healthy human subjects. We found clear in vivo and in vitro evidence that SRIs may alter T cell responsiveness.

## Materials and methods

### Reagents

Citalopram, sertraline, fluvoxamine and venlafaxine were purchased from Sigma Aldrich (St-Louis, MO, USA). Paroxetine was purchased from Fagron (Nieuwerkerk a/d IJssel, The Netherlands), and fluoxetine from ABC chemicals (Wouters-Brakel, Belgium). For animal experiments, fluoxetine was dissolved in PBS. For in vitro experiments, the drugs were diluted in RPMI-1640, supplemented with 10% heat-inactivated fetal bovine serum, 1% glutamine and 1% penicillin/streptomycin (100 U/ml penicillin G; 100 μg/ml streptomycin). All cell culture reagents were purchased from Invitrogen (Carlsbad, CA, USA).

### Animals

Ten- to 12-week old female AKR (H-2^k^, Thy1.1, Mls1a/2b) mice were used as recipients and 6- to 8-week old C3H (H-2^k^, Thy1.2, Mls1b/2a) mice as donors. Mice were purchased from Harlan BV, The Netherlands. Recipients were housed in groups of four or five in individually ventilated cages. Animals were fed standardised pellet chow and water, decontaminated by UV irradiation or by acidification. All experiments were approved by the Ethical Committee for Animal Science of the University of Leuven.

### GvHD model and SRI treatment

Bone marrow (BM) cells were obtained by flushing RPMI containing 1% heparin through the shafts of the femurs and tibia of C3H donor mice. T cell depletion was performed using cytotoxic complement-fixing anti-Thy1.2 antibody and low toxic rabbit complement (Serotec, Oxford, United Kingdom) as described previously (Billiau et al. 2002). AKR recipient mice received a single dose of 9.5 Gy total body irradiation on day -1. Within 24h after completion of irradiation, either 5×10^6^ T cell depleted BM (BMT only) or 5×10^6^ T cell depleted BM in combination with 50×10^6^ spleen cells (BMT + SPL) were injected into a tail vein in a total volume of 250 μl. Recipient mice were treated with 20 mg/kg fluoxetine IP 2×/day at the day of transplantation, 1×/day for the following 11 days, and 3×/week for the rest of the experiment. Control animals received vehicle (PBS) only. The ‘BMT only’ group did not receive any treatment.

### GvHD scoring

Animals were inspected on a daily basis. Signs of GvHD typically observed in this model are ruffled fur and hunched posture, lethargy, inflammation of the eyes, weight loss and diarrhea (Sefrioui et al. 2000; Billiau et al. 2002). The mice were weighed and scored for GvHD once weekly, using a GvHD scoring system previously described in this model (Fevery et al. 2007). Each parameter received a score as followed: 0 = normal, 1 = mild, 2 = moderate, 3 = severe. For body weight, the following scoring system was used: 0 = 100–90%, 1 = 90–80%, 2 = 80–70%, 3 = <70% of initial body weight. The maximum score was 15. Mice that succumbed to GvHD received the maximum score of 15.

### Donor T cell chimerism and host-reactive donor T cell frequency

The percentage of donor T lymphocytes in peripheral blood was determined by flow cytometry, based on the differential expression of Thy1.2 (donor) and Thy1.1 (recipient). Following red blood cell lysis with NH_4_Cl, cells were labelled with FITC- or PE-conjugated anti-Thy1.1 and anti-Thy1.2 (Serotec, Oxford, UK). The frequency of host-reactive TCR-Vβ6+ T cells was determined as a parameter of in vivo alloreactive T cell expansion (Billiau et al. 2002; Sefrioui et al. 2000). Cells were labelled with APC-, PE- or PerCP-conjugated antibodies against CD3 and TCR-Vβ6 (BD Biosciences, Erembodegem, Belgium).

### In vitro apoptosis assay

Human peripheral blood mononuclear cells (PBMCs) from six healthy volunteers were obtained by Ficoll density centrifugation. T cells were isolated from PBMCs using a human T cell enrichment kit (STEMCELL technologies, Vancouver, Canada) according to the manufacturer’s instructions. T cell purity was determined by staining with anti-CD3 PECy5 and flow cytometric analysis and was in each experiment greater than 97%. Anti-CD3/CD28 beads (Dynal Biotech ASA, Oslo, Norway) were added in a 1:1 ratio and 2×10^5^ cells were seeded per well in a total volume of 200 μl. After a 24h-incubation in the presence of SRIs, cells were stained with 0.5 μl annexin V-FITC and 10 μl propidium iodide (PI) (BD Pharmingen, San Diego, CA, USA). Activation status was determined by staining with anti-CD69 PECy7 (eBiosciences, San Diego, CA, USA).

### In vitro proliferation assay

Isolated PBMCs from six healthy volunteers were stained with 10 μM carboxy-fluorescein diacetate succinimidyl ester (CFSE) (Invitrogen, Carlsbad, CA, USA) according to the manufacturer’s instructions. Subsequently, cells were activated with 4×10^5^ anti-CD3/CD28 beads per 10^6^ PBMCs. 25×10^4^ PBMCs were seeded per well in a total volume of 250 μl and incubated for 6 days in the presence of SRIs. Thereafter, cells were stained with anti-CD3 PECy5 (eBiosciences, San Diego, CA, USA). All analyses were performed on a Cytomics FC500 flow cytometer (Beckman Coulter, Miami Florida). Dead cells were excluded based on FSC-SSC properties. For CD3+ T cells, a proliferation index was calculated according to the following formula (Roederer 2011):

$$ \mathrm{Proliferation}\;\mathrm{i}\mathrm{n}\mathrm{dex}=\frac{{\sum {_0^i{N_i}} }}{{\sum {_0^i\frac{{{N_i}}}{{{2^i}}}} }}\mathrm{ith}\;\mathrm{i}=\mathrm{generation}\;\mathrm{number}\;\left( {0\;\mathrm{i}\mathrm{s}\;\mathrm{the}\;\mathrm{undivided}\;\mathrm{population}} \right)\;\mathrm{and}\;\mathrm{Ni}=\mathrm{the}\;\mathrm{number}\;\mathrm{of}\;\mathrm{events}\;\mathrm{i}\mathrm{n}\;\mathrm{generation}\;\mathrm{i}. $$

All in vitro experiments were approved by the Ethical Committee of the Ghent University Hospital.

### Statistics

In vivo experiments. The Gehan-Breslow-Wilcoxon test was used to estimate the level of significance of the difference in survival between treatment groups. The Wilcoxon signed ranks test was used to identify statistically significant differences for GvHD scores and flow cytometry data between treatment groups. In vitro experiments. Wilcoxon signed ranks tests were used to identify statistically significant differences between treatment and control, and between activated and resting T cells. Results were considered statistically significant if one-tailed *p*-values were <0.05.

## Results

### Fluoxetine delays the onset and attenuates the severity of GvHD

An established model of acute GvHD in a MHC-matched miHA-mismatched mouse strain combination was used (Sefrioui et al. 2000; Billiau et al. 2002). AKR recipient mice carry the Mtv-7 retrovirus which encodes the Mls-1 antigen, leading to deletion of TCR-Vβ6+ T cells. C3H donor mice do not carry the Mtv-7 virus and therefore TCR-Vβ6+ T cells are retained in these mice. In this model, donor and recipient mice differ in their expression of the Mtv7-genome, which has been shown to be associated with a highly increased rate and severity of GvHD (Miconnet et al. 1995).

An IP dose of 20 mg/kg fluoxetine was administered twice at the day of transplantation in order to achieve high enough plasma levels to prevent alloreactive T cells to initiate an immune reaction. To prevent alloreactivity during the immediate posttransplant period (day 2–12), fluoxetine was administered 1×/day. In this period, the frequency and severity of GvHD is higher, possibly because of overstimulation of host-reactive T cells by the remnants of the cytokine storm elicited by the conditioning regimen (Antin and Ferrara 1992). In our murine model, cytokine mRNA expression is diminished by day ten after irradiation (Billiau et al. 2001). During the rest of the experiment, a maintenance dose was given 3×/week. A 20 mg/kg IP dose was chosen as this dose gives rise to a plasma concentration of 4 μM (Holladay et al. 1997), a concentration that showed optimal anti-proliferative effect in preliminary murine mixed lymphocyte reactions without inducing cytotoxicity (data not shown).

**Fig. 1:**
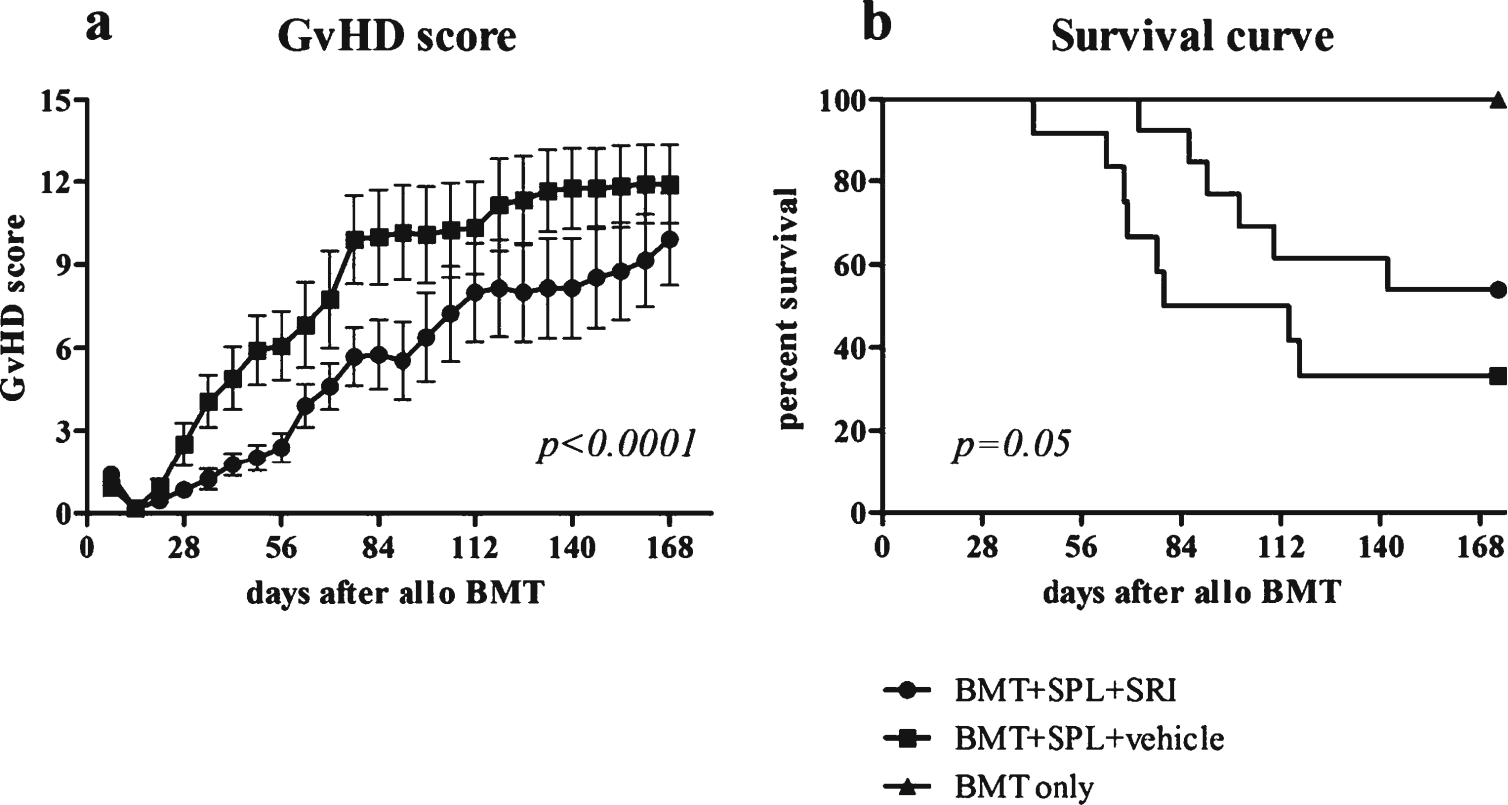
Effect of fluoxetine on GvHD score and survival. AKR mice were irradiated with 9.5 Gy on day -1 and transplanted with 5×10^6^ C3H BM only or together with 50×10^6^ SPL cells on day 0. Mice were treated with 20 mg/kg IP fluoxetine or vehicle 2×/day on the day of transplantation, 1×/day for the following 11 days and 3×/week for the rest of the experiment. **a** mean ± SEM GvHD scores from BMT+SPL+SRI group (*n*=13) and BMT+SPL+vehicle group (*n*=12). GvHD score was based on five parameters, each receiving a score of 0–3: ruffled fur and hunched back, inflammation of the eyes, weight, diarrhea and lethargy. **b** Survival curve. Results are the percentage survival from BMT+SPL+SRI (*n*=13), BMT+SPL+vehicle (*n*=12) and BMT only (*n*=5) groups. Results are pooled data from two different experiments

Throughout the course of the experiment, mice were observed daily for clinical symptoms of GvHD and the GvHD score was recorded weekly. Mice that were treated with vehicle developed typical symptoms of GvHD (score > 2) after 4 weeks, whereas fluoxetine-treated mice only showed clinical signs of illness after 8 weeks (Fig. 1a). Although fluoxetine-treated mice did develop clinical symptoms in the course of the experiment, GvHD was less severe in this group compared to vehicle-treated mice (*p*<0.0001). Control mice receiving either BMT+SRI (*n*=6), BMT+vehicle (*n*=7) or BMT only (*n*=5) did not develop clinical signs of GvHD (data not shown).

### Fluoxetine reduces GvHD lethality

In Fig. 1b, the survival of AKR mice after transplantation of 5×10^6^ C3H BMT only or together with 50×10^6^ SPL cells and treated with 20 mg/kg fluoxetine or vehicle is shown. Whereas only 4/12 (33.3%) animals from the vehicle-treated group survived 6 months after transplantation, 7/13 (53.8%) mice survived in the fluoxetine-treated group (*p*=0.05). Control mice receiving either BMT+SRI (*n*=6) (data not shown), BMT+vehicle (*n*=7) (data not shown) or BMT only (*n*=5) all survived until the end of the experiment.

### Fluoxetine does not interfere with engraftment of cells

**Fig. 2:**
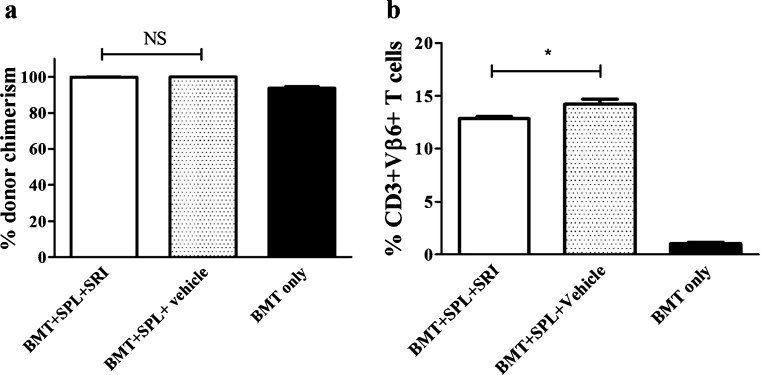
Donor chimerism and alloreactivity. **a** Percentage donor T cell chimerism in peripheral blood lymphocytes, determined by Thy1.1 (recipient) and Thy1.2 (donor) positivity. **b** percentage alloreactive CD3+Vβ6+ T cells. Results are mean ± SEM from 5 animals in BMT+SPL+SRI and BMT only group and from 4 animals in BMT+SPL+vehicle group. Statistically significant differences are depicted with * (one-tailed *p*<0.05). NS = not significant

In order to determine whether fluoxetine interferes with the engraftment of the allogeneic cells, peripheral blood donor T cell chimerism was determined at week 8 after transplantation, a time point at which donor T cell chimerism – in the absence of GvHD - can be expected to be near-complete (Billiau et al. 2002). Thy1.2 and Thy1.1 expression was used to discriminate between donor- and recipient-derived lymphocytes, respectively. Both fluoxetine-treated and vehicle-treated mice showed a donor T cell chimerism of more than 99%, indicating that the efficiency of the stem cell transplantation was equal in both groups and was not negatively influenced by fluoxetine. Consistent with previous work in this model, donor T cell chimerism of the ‘BMT only’ group was around 90% (Fig. 2a).

### Fluoxetine reduces the expansion of donor-type host-reactive T cells

In murine GvHD models involving Mls-disparate mouse strains, GvHD has been shown to be associated with expansion of donor-type T cells bearing TCR Vβ chains that are specific for host-type Mls antigens (Sefrioui et al. 2000; Johnson et al. 1995; Billiau et al. 2002). Accordingly, GvHD in C3H-AKR chimeras is associated with an expansion of TCR-Vβ6+ T cells (Sefrioui et al. 2000; Billiau et al. 2002). In order to determine whether fluoxetine suppressed the expansion of these host-reactive T cells, we determined the frequency of CD3+Vβ6+ T cells in peripheral blood of chimeras at week 8 after bone marrow transplantation, a time point at which control mice showed clear GvHD whereas SRI-treated mice were still free of GvHD clinical symptoms (score ≤ 2). The results are shown in Fig. 2b. Fluoxetine-treated mice showed a significantly lower percentage of peripheral blood CD3+Vβ6+ T cells than vehicle-treated mice (*p*=0.016), indicating that the beneficial effect of fluoxetine on GvHD is indeed associated with a reduced expansion of host-reactive T cells.

### Activated human T cells are more sensitive to SRI-induced apoptosis than resting T cells

We next explored whether the observed effect of SRIs on the T cell response in murine GvHD can be due to a direct effect on T cells. To this end we investigated the viability of both resting and activated T cells, obtained from 6 healthy human volunteers, when exposed to SRIs in vitro. In this study, we did not only investigate the effects of fluoxetine, but incorporated the most frequently used SRIs (paroxetine, sertraline, citalopram, fluvoxamine and venlafaxine). T cells were stimulated using anti-CD3/CD28 beads and the activation status of the cells was confirmed by the expression of CD69. More than 80% of the T cells were CD69 positive after a 24h-incubation with anti-CD3/CD28 beads (‘activated T cells’). In contrast, less than 2% of the T cells that were not stimulated expressed CD69 (‘resting T cells’).

To detect apoptotic cells, annexin V and PI staining was performed. PI staining correlated well with annexin V data (correlation coefficients were typically >0.99) and therefore, only annexin V data were further used for data analysis. The mean percentage annexin V+ cells in control samples (*n*=6) was 4.05 ± 1.72% for resting T cells and 5.99 ± 4.65% for activated T cells. In order to compare apoptosis rates in resting and activated T cells, annexin V+ percentages obtained in control samples were subtracted from the individual percentages determined in the test samples. Thus, differences in SRI-induced apoptosis found between resting and activated T cells cannot be ascribed to differences in basal apoptosis.

Detailed analysis of the annexin V+ T cell percentages revealed that paroxetine (*p*= 0.016) and sertraline (*p*=0.031) significantly induced apoptosis in activated T cells at 5 μM, while for the other SRIs, no apoptosis could be detected at this concentration. For fluoxetine on the other hand, apoptosis could be detected at concentrations of 10 μM or higher in activated T cells (*p*=0.016). Fluvoxamine showed a similar effect, but at a higher concentration range: apoptosis started to appear at concentrations of 50 μM (*p*=0.016). Citalopram only induced a slight increase in apoptotic cells, and statistical significance was reached at 100 μM only (*p*=0.016). No apoptosis could be detected after treatment with venlafaxine at concentrations up to 100 μM.

**Fig. 3:**
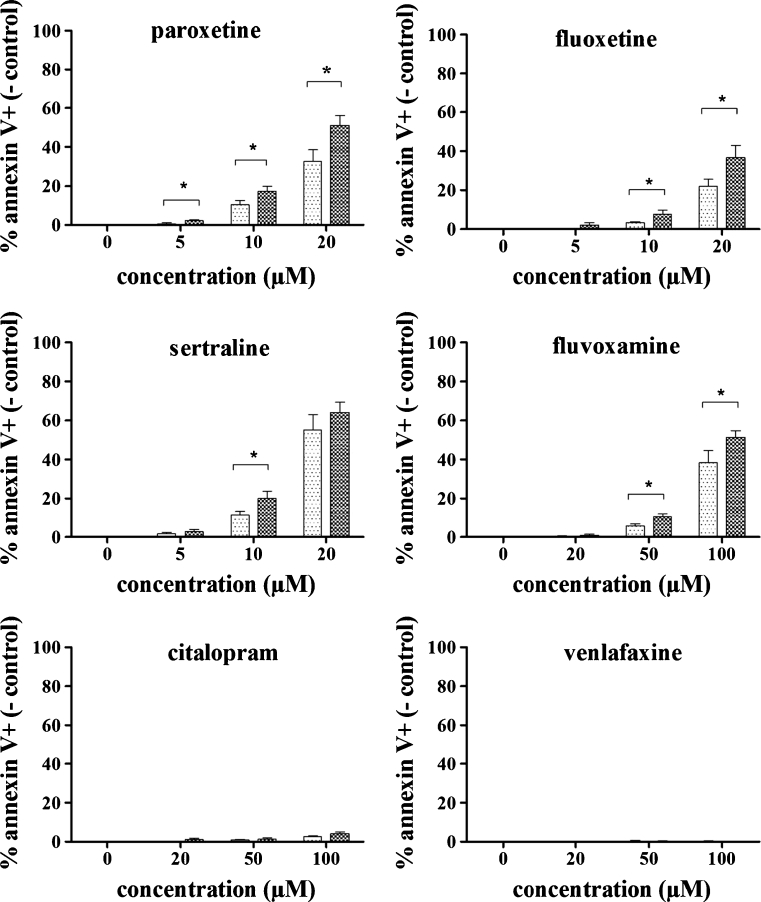
Apoptotic effect of SRIs on activated and resting T cells. In vitro activated and resting T cells were incubated for 24h with 0–20 μM paroxetine, 0–20 μM fluoxetine, 0–20 μM sertraline, 0–100 μM fluvoxamine, 0–100 μM citalopram, 0–100 μM venlafaxine and subsequently analyzed by annexin V staining. Mean ± SEM percentages of annexin V+ cells (- control) in activated and resting T cells are shown (*n*=6). () = T cells activated with anti-CD3/CD28 beads in a 1:1 bead:cell ratio; () = resting T cells. Statistically significant differences between activated and resting T cells are depicted with * (one-tailed *p*<0.05).

Interestingly, activated T cells were more sensitive to the apoptotic effect compared to resting T cells (Fig. 3). Paroxetine, fluoxetine, sertraline and fluvoxamine induced significantly more apoptosis in activated T cells than in resting T cells. For paroxetine, already the lowest tested concentration (5 μM) induced significantly more apoptosis in the activated cells (*p*=0.031). This difference was maintained with higher concentrations (10 μM, *p*=0.016 and 20 μM, *p*=0.016). For fluoxetine (*p*=0.016) and sertraline (*p*=0.031), a significantly higher effect could be detected in the activated T cells at 10 μM. Fluoxetine maintained this significantly higher induction of apoptosis at 20 μM (*p*=0.031). In contrast, sertraline did not exert a differential effect on activated and resting T cells at the highest concentration tested (20 μM). The absence of a significant difference at this concentration might be due to an increased cytotoxicity of this high concentration of sertraline. Fluvoxamine induced significantly more apoptosis in the activated T cells at the 2 highest concentrations tested (50 μM, *p*=0.016 and 100 μM, *p*=0.016). Citalopram did not exert a differential effect on activated versus resting T cells, however this may be due to the fact that citalopram only induced a very low percentage of apoptosis in both cell populations. No apoptosis could be detected after treatment with venlafaxine in concentrations up to 100 μM.

### SRIs can inhibit T cell proliferation at concentrations that do not affect T cell viability

In order to evaluate the effect of SRIs on T cell proliferation, PBMCs from healthy volunteers were labelled with CFSE, activated with anti-CD3/CD28 beads and incubated for 6 days in the presence of SRIs. The amount of T cells in each cell cycle was determined by flow cytometry and the results were expressed as a proliferation index.

**Fig. 4:**
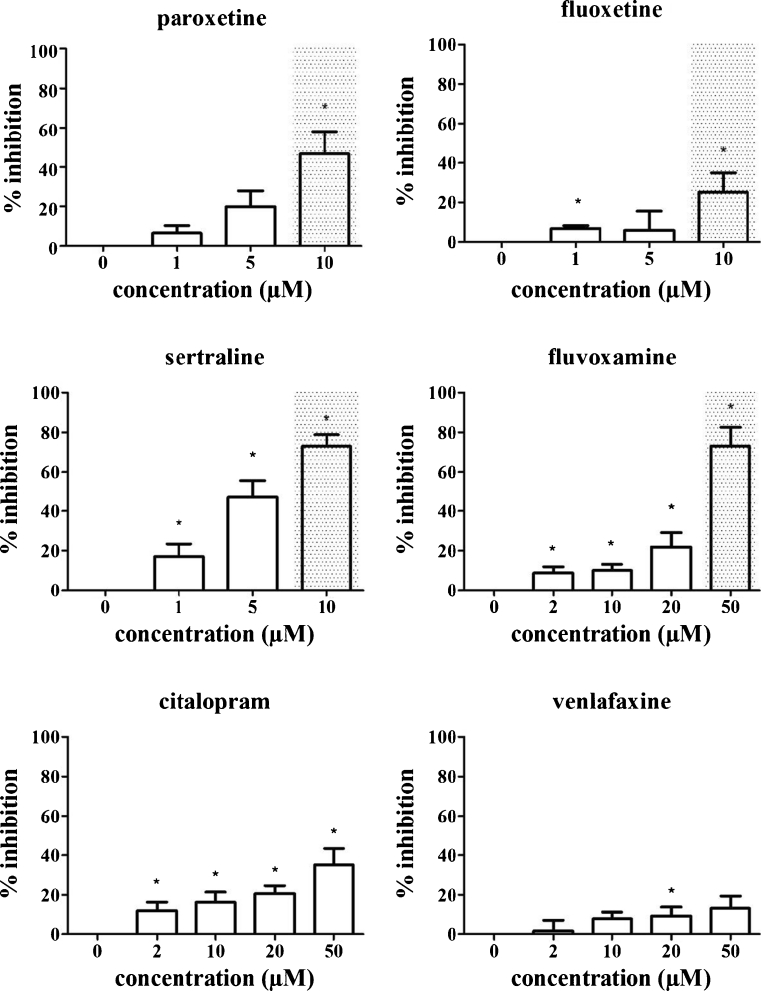
Inhibition of T cell proliferation by SRIs. PBMCs were labelled with CFSE, activated with 4×10^5^ anti-CD3/CD28 beads per 10^6^ cells and incubated in the presence of 0–10 μM paroxetine, 0–10 μM fluoxetine, 0–10 μM sertraline, 0–50 μM fluvoxamine, 0–50 μM citalopram, 0–50 μM venlafaxine for 6 days. Viable CD3+ cells were gated and proliferation indices were calculated based on the number of cells in each division peak. Values are expressed as % inhibition in comparison to control cells not exposed to SRIs. Mean ± SEM % inhibition of 6 individual experiments are shown. Concentrations that induce more than 5% apoptosis in activated and/or resting T cells are displayed with gray background. Statistically significant differences in proliferation indices compared to control cells not exposed to SRIs are depicted with * (one-tailed *p*<0.05)

All SRIs tested decreased the proliferation index in a concentration-dependent manner (Fig. 4). Paroxetine exerted an anti-proliferative effect at 10 μM (*p*=0.016). Fluoxetine and sertraline significantly decreased the proliferation index at concentrations as low as 1 μM (*p*=0.018 and *p*=0.047 respectively). Fluvoxamine and citalopram significantly decreased T cell proliferation at 2 μM (lowest dose tested, *p*=0.029 and *p*=0.016 respectively). For venlafaxine, higher doses were needed in order to reduce T cell proliferation: a significant decrease for venlafaxine was detected only at 20 μM (*p*=0.047). Importantly, the SRI concentrations needed to reduce T cell proliferation were, except for paroxetine, below those inducing apoptosis in activated and/or resting T cells (gray background in Fig. 4).

The strongest decrease in T cell proliferation was induced by sertraline. Whereas a 5 μM concentration only slightly affected the viability of resting T cells (an increase of 1.78 ± 1.38% annexin V+ cells was observed as compared to controls), this concentration also dramatically reduced the proliferation of activated T cells (a mean reduction of 39.4% was found). At even higher concentrations, proliferation was almost completely inhibited, but in this concentration range, also the viability of both resting and activated T cells was affected.

We clearly documented that fluoxetine treatment is able to suppress in vivo allo-activated T cell responses and suppress clinical GvHD in a mouse model. Acute GvHD was induced in C3H → AKR radiation bone marrow chimeras, according to a previously validated model (Sefrioui et al. 2000) and mice were treated either with a high-dose fluoxetine regimen, or with vehicle. Further, we provided a comprehensive study comparing the anti-proliferative and pro-apoptotic effects of all available SRIs in both activated and resting human lymphocytes. We clearly demonstrated that in vitro exposure of human T cells to SRIs affects their responsiveness and viability.

## Discussion

Several lines of evidence exist that SRIs exert an influence on the immune system. First, in vitro studies have shown a suppressive effect of SRIs on both rat and human lymphocyte proliferation and viability (Pellegrino and Bayer 2000; Taler et al. 2007). Second, animal studies have demonstrated that SRIs can attenuate symptoms and inflammatory activity in selected autoimmune disorders, such as experimental autoimmune encephalomyelitis (Taler et al. 2011; Vollmar et al. 2009; Yuan et al. 2012), collagen-induced arthritis (Sacre et al. 2010), septic shock and allergic asthma (Roumestan et al. 2007) and contact hypersensitivity reaction (Kubera et al. 2012). These studies suggest that SRIs might be beneficial in the treatment of autoimmune pathologies. Third, clinical case reports indicate that SRIs, when administered in high doses, may influence immune function. For instance, Reed and Glick report a reactivation of herpes simplex virus in patients receiving high doses of fluoxetine (Reed and Glick 1991). Also, a case of recurring sinusitis was associated with venlafaxine use (Denys et al. 2002).

The above mentioned papers indicate that SRIs might interfere with pathologically activated autoreactive T cells. Here, we investigated the role of SRIs in allo-antigen activated T cells in the course of acute GvHD. Acute GvHD after allogeneic haematopoietic stem cell transplantation (alloHSCT) is a cause of extensive morbidity and mortality. We used a murine MHC-matched, miHA-mismatched bone marrow transplantation model to investigate whether fluoxetine could reduce GvHD. The MHC-matched, miHA-mismatched model was chosen in analogy with the human situation where over 50% of alloHSCT patients receive an HLA-matched graft (Hurley et al. 2003). In our study, fluoxetine was found to significantly delay and reduce clinical symptoms, without interfering with the reconstitution of the hematopoietic compartment. An improvement in survival rate was also noted. Furthermore, the percentage of CD3+Vβ6+ T cells was significantly reduced by fluoxetine, consistent with an inhibitory effect of this compound on the expansion of alloreactive T cells. This leads to the assumption that fluoxetine, and possibly also other SRIs can have a beneficial effect on the outcome of acute GvHD.

In our murine model, a 20 mg/kg fluoxetine dose was sufficient to delay and reduce clinical symptoms of GvHD. In comparison, fluoxetine doses used in mice to obtain an ‘antidepressive’ effect are around 10–18 mg/kg (Dulawa et al. 2004). A single IP dose of 20 mg/kg fluoxetine in mice gives rise to a serum concentration around 4 μM, measured 30 min after administration (Holladay et al. 1997). Although plasma concentrations of SRIs in depressive patients are below 1 μM (Holladay et al. 1997), SRIs are known to have a wide therapeutic-toxic range in humans and higher dosing may be achieved without serious adverse effects. Fatal overdosing with SRIs is very rare and doses up to 30 times the normal daily doses either do not cause any adverse effects or only minor effects (Barbey and Roose 1998). Therefore, plasma concentrations needed for immunomodulation are expected to be feasible.

Several papers reported on the effect of SRIs on the immune response, but large differences are seen in the type of SRI, the experimental setup, the test species and the detection methods. Therefore, the need emerged to correlate all these previous findings and extend them in order to obtain one standardized study that gives an overview of the effect of all clinically available SRIs on both activated and resting T cells. This study compared the in vitro immunomodulatory effects of five selective SRIs (paroxetine, fluoxetine, sertraline, fluvoxamine, citalopram) and one serotonin and noradrenalin reuptake inhibitor (venlafaxine). We incubated human PBMCs or purified T cells with SRIs in vitro and determined the effect on viability and proliferation. SRIs were shown to exert direct in vitro effects on the viability and proliferation of T cells. Paroxetine, fluoxetine, sertraline, fluvoxamine and citalopram were found to induce apoptosis in activated T cells, and this pro-apoptotic effect was significantly lower in resting T cells. For citalopram, only a slight increase in apoptosis could be detected and no differential effect on activated versus resting T cells was found. However, it should be noted that others showed a substantial pro-apoptotic effect in resting T cells for citalopram at 180 μM (Xia et al. 1997). Therefore, it cannot be ruled out that citalopram exerts a differential effect at concentrations higher than 100 μM. Venlafaxine did not induce apoptosis in both activated and resting T cells at concentrations up to 100 μM.

Further, all SRIs were found to reduce T cell proliferation in a concentration-dependent manner, at concentrations below those inducing apoptosis (except for paroxetine, which inhibited T cell proliferation and viability at the same concentration). Since the concentrations needed to significantly reduce T cell proliferation are substantially lower than those affecting resting T cell viability, SRIs could be used to suppress proliferation of pathologically activated T cells while at the same time the repertoire of resting T cells remains unaffected and preserves the capability of reacting to pathogens and cancer cells at later stages. Moreover, since sertraline produced a strong anti-proliferative effect while at the same time retaining resting T cell viability, these data are also promising for the use of sertraline in GvHD. Further research on this SRI is therefore requested.

In the central nervous system, SRIs inhibit reuptake of serotonin through the serotonin transporter (SERT) in the presynaptic neuron, resulting in increased synaptic serotonin concentrations (Anderson et al. 2002). Although SERT expression has also been shown in lymphocytes (Faraj et al. 1994; Fazzino et al. 2008), it is doubted that the immunosuppressive effects of SRIs are mediated through the serotonergic system (Cloonan et al. 2010; Diamond et al. 2006). On the other hand, it has been suggested that the immunological effects of SRIs are due to induced changes in several signaling pathways. SRIs have been demonstrated to interfere with the activation of the cAMP-dependent protein kinase A (PKA) pathway and the activation of protein kinase C (PKC), as well as with the influx of Ca^2+^ (Maes et al. 2005; Edgar et al. 1998; Edgar et al. 1999; Kenis et al. 2003). Furthermore, SRI-mediated induction of apoptosis was accompanied by activation of the MAPK signaling pathway and downregulation of the anti-apoptotic factor bcl-2 (Taler et al. 2008). Finally, it has been suggested that triggering of SERT itself can induce changes in downstream signaling pathways (Serafeim et al. 2002), thus linking the known affinity of SRIs for SERT with the observed changes in signaling pathways. However, the exact mechanism through which SRIs induce immunosuppression requires further investigation.

Our in vitro data indicate that not all SRIs have the same magnitude of effect in lymphocytes. Whereas paroxetine, fluoxetine and sertraline exert immunosuppressive effects at concentrations below 10 μM, fluvoxamine, citalopram and venlafaxine only exert immunosuppressive effects at 50 μM or higher. Since the mechanism through which SRIs induce immunosuppression is not fully understood, it is difficult to interpret differences in SRIs that might explain the observed differences in immunological effects. However, assuming that triggering of SERT is important for the observed effects, the difference in immunological effects might be explained by the different affinity of SRIs for SERT. The three SRIs that exert the strongest immunosuppressive effects (paroxetine, fluoxetine and sertraline), also have the highest affinity for SERT (Kd < 1 nM). The SRIs that show less or no immunosuppressive action, fluvoxamine, citalopram and venlafaxine, have only moderate affinity for SERT (1 nM < Kd < 10 nM) (Tatsumi et al. 1997). Possibly, the lack of effect for venlafaxine might as well be explained by the fact that venlafaxine is a mixed serotonin and noradrenalin reuptake inhibitor, whereas the other tested SRIs are selective for serotonin. In the case of citalopram, it is known that the R-enantiomer induces an allosteric modification in SERT, thereby reducing the binding capacity of the active S-enantiomer escitalopram (Sanchez 2006). Possibly, the lack of immunosuppressive effect seen with the racemic mixture citalopram is due to the presence of the R-enantiomer.

In conclusion, this study shows that fluoxetine can delay and reduce clinical symptoms of experimental GvHD, along with an inhibition of the expansion of alloreactive T cells. Data on T cells from healthy human subjects show that this effect may be attributed to a direct anti-proliferative and pro-apoptotic effect. Given the similar T cell immunosuppressive effects of other SRIs in vitro, the potential application of these compounds in GvHD should be investigated. Together with prior studies in EAE and CIA, our data from a GvHD mouse model support the exploration of the therapeutic value of SRI-induced T cell suppression in GvHD and other immune-mediated disorders. Moreover, the data underscore the need for further research into the potential immunomodulatory effects of the therapeutic use of SRIs in humans.
